# Virtual Reality to Reduce Preprocedural Anxiety During Invasive Coronary Angiography: The VR InCard Trial

**DOI:** 10.1016/j.jacadv.2025.101976

**Published:** 2025-07-18

**Authors:** Tjitske D. Groenveld, Esther H.W. Breunissen, Judith L. Bonnes, Linda Garms, Naomi Scholten, Aysun Cetinyurek-Yavuz, Lokien X. van Nunen, Marleen X. van Wely, Cyril Camaro, Tim J.F. ten Cate, Aukelien C. Dimitriu-Leen, Niels van Royen, Robert Jan M. van Geuns, Dominique V.M. Verhaert, Harry van Goor, Peter Damman

**Affiliations:** a Department of Surgery, Radboud University Medical Center, Nijmegen, the Netherlands; b Department of Cardiology, Radboud University Medical Center, Nijmegen, the Netherlands; c Department of Cardiology, Cardiovascular Research Institute Maastricht (CARIM), Maastricht University Medical Center (MUMC+), Maastricht, the Netherlands

**Keywords:** anxiety, coronary angiography, nonpharmacological therapy, periprocedural anxiety, virtual reality

## Abstract

**Background:**

Anxiety affects one-third of patients undergoing invasive coronary angiography (ICA). Benzodiazepines are commonly administered but have modest efficacy. Virtual reality (VR) therapy is promising in reducing anxiety around various procedures.

**Objectives:**

This study aimed to evaluate the effect of VR on preprocedural anxiety in patients undergoing ICA.

**Methods:**

This randomized controlled trial included adults undergoing ICA for chronic coronary syndrome, coronary vasomotor function testing, or non-ST-segment elevation acute coronary syndrome (NSTE-ACS) with a numeric rating scale (NRS) anxiety score of 4 or more. The control group received usual care with benzodiazepines if needed. The intervention group additionally received 2 VR therapysessions of approximately 20 and 10 minutes before ICA. The primary outcome was preprocedural NRS anxiety. Secondary outcomes included NRS anxiety at other timepoints, physiological stress parameters, and patient- and provider-reported outcome measures. Subgroup analyses were conducted by sex and ICA indication.

**Results:**

A total of 99 patients were included, 47 in the intervention group and 52 in the control group. No difference was observed in preprocedural anxiety. After adjusting for baseline anxiety, the intervention group showed a significant reduction in preprocedural NRS anxiety (mean difference: 0.9; 95% CI: 0.2-1.6; *P* = 0.010). No significant differences were observed in secondary outcomes. Patients undergoing ICA for NSTE-ACS experienced the greatest effect.

**Conclusions:**

VR therapy did not reduce preprocedural anxiety in patients undergoing ICA. After adjusting for baseline anxiety, VR therapy significantly reduced preprocedural anxiety, with the most pronounced effect in patients undergoing ICA for NSTE-ACS. VR therapy presents a viable nonpharmacological complement to traditional treatments for procedure-related anxiety.

Invasive coronary angiography (ICA) with potential percutaneous intervention or coronary function testing is commonly performed to assess acute or chronic ischemic heart disease. It is an essential procedure for the diagnosis and treatment of coronary artery disease but can cause high anxiety levels in patients.^1^ Up to one-third of patients undergoing ICA experience anxiety, with the highest levels occurring immediately before the procedure.^1^^,^^2^ Patients' concerns are mainly centered on the potential diagnostic outcomes, possible procedural risks, unfamiliarity with the hospital environment, and fear of the unknown.^1^ Hearing negative experiences of other patients who underwent a similar procedure or not being appropriately introduced to the treatment team beforehand can additionally trigger concerns and anxiety.^3^

Anxiety compromises patient comfort due to activation of the sympathetic nervous system, resulting in increased heart rate and changes in vascular tone and increased susceptibility to rhythm disturbances.^3^^,^^4^ In addition, higher levels of anxiety around ICA procedures are associated with higher levels of depression and anxiety afterward.^5^^,^^6^ In turn, high levels of anxiety and depression are associated with worse physiological outcomes.^3^^,^^7^ It is therefore important to identify effective strategies for alleviating anxiety in patients undergoing ICA.

The effectiveness of benzodiazepines, commonly administered preprocedurally to reduce anxiety, has been shown to be very modest.^8^^,^^9^ In addition, these drugs can cause undesirable side effects such as drowsiness, respiratory depression, and adverse drug interactions.^10^ In some patient groups or procedures, benzodiazepines are contraindicated. For example, benzodiazepines can potentially influence the diagnostic outcome in patients who undergo coronary vasomotor function testing and are preferably avoided.

Nonpharmacological alternatives for anxiolysis include preprocedural education and relaxing interventions.^10^ Educational videos have been shown to improve patient comprehension and compliance with the procedure; however, patients often still experience significant anxiety.^11^ Music therapy has demonstrated effectiveness in reducing anxiety, pain, and the need for periprocedural sedatives.^12^ However, patients remain aware of their surroundings, which can continue to be a source of anxiety. Virtual reality (VR) therapy is a novel treatment that combines music therapy with the immersion of patients in a comforting virtual environment. VR distraction and hypnosis have been shown to effectively reduce pain and anxiety associated with other procedures like burn wound care or venipuncture.^13^^,^^14^ VR distraction engages patients in captivating virtual environments or interactive games, creating a comforting experience that redirects their attention away from the experienced anxiety.^14^, ^15^, ^16^ VR hypnosis utilizes immersive experiences to focus patients' attention on positive aspects, enhancing the patient's ability to manage stress, anxiety, and pain through guided imagery and narration.^17^

In the current study, we assessed the effectiveness of VR therapy in reducing periprocedural anxiety in an all-comer population of patients undergoing ICA. This approach aimed to evaluate the practical effectiveness of VR therapy in a real-world clinical setting and assess its possible clinical impact.

## Methods

### Study design

This single-center open-label randomized controlled trial assessed the effect of VR therapy on periprocedural anxiety in patients undergoing ICA. The trial was performed at the cardiology department of the Radboud University Medical Center Nijmegen, the Netherlands. The design of this trial has been previously outlined in detail.^18^ This trial was registered at ClinicalTrials.gov (NCT06215456) and conducted according to the principles of the Helsinki Declaration and in compliance with Dutch guidelines, regulations, and acts (Medical Research involving Human Subjects Act, WMO). Results were reported following CONSORT (Consolidated Standards of Reporting Trials) guidelines.^19^

### Study population

Patients were eligible if they were undergoing ICA for chronic coronary syndrome, non-ST-segment elevation-acute coronary syndrome (NSTE-ACS), coronary vasomotor function testing (CFT), or preparation for cardiovascular surgery. Further inclusion criteria were: 1) patients aged 16 years or older; 2) numeric rating scale (NRS) anxiety score of 4 or more upon arrival at the day care unit; 3) ability to understand and speak the Dutch language; and 4) willingness and ability to comply with the study protocol. An anxiety score of 4 or higher was selected based on the local protocol, which advises offering benzodiazepines to patients with scores above this threshold. Exclusion criteria were: 1) history of dementia; 2) severe hearing/visual impairment that is not corrected; and 3) history of depression or generalized anxiety disorder.

### Intervention

The control group received standard care. At the daycare unit, preprocedural benzodiazepines (standard oxazepam 10 mg, oral) were administered through shared decision-making with the patient and nurse, based on patient preference, comorbidity, and medication use according to local practice. In the catheterization room, benzodiazepines (diazepam 2.5 mg, intravenous) were routinely administered. However, benzodiazepines were withheld in patients scheduled for CFT. As part of standard care, all patients undergoing elective ICA received patient education through oral information, an information letter, and an online information video. Patients undergoing ICA for NSTE-ACS received only oral information following standard care.

Patients in the intervention group received VR therapy, including VR hypnosis and distraction. Patients received 2 VR hypnosis sessions in addition to standard care, directly preprocedural and while arterial access was obtained. Additionally, patients were allowed to use VR distraction at their own discretion between admission and the first VR hypnosis session, when scheduling allowed. The first VR hypnosis session lasted approximately 20 minutes and was administered at the daycare unit 20 minutes prior to the procedure. The second VR hypnosis session was administered in the cardiac catheterization room after introduction of the treatment team, approximately 5 minutes prior to arterial puncture. This second session was ended when the coronary catheter was positioned at the aortic root. At this point, the VR headset was removed, and the rest of the procedure continued following standard practice. The duration of the second session depended on the duration of the process of obtaining arterial access.

VR therapy was applied using a head-mounted display, the PICO G2 4K. For VR hypnosis, the application “HypnoVR” was used. This app contains several narratives directing the patients away from their surroundings, integrating sequences of cardiac coherence breathing while guiding patients virtually through a natural environment (Supplemental Figure 1). “SyncVR Relax & Distract” (SyncVR Medical) was used for VR distraction. Patients were allowed to choose from a wide range of relaxation games, videos, and exercises, each with a duration of 5 to 20 minutes.

### Data collection and outcomes

Patient characteristics were registered at baseline, including age, sex, previous angiographies, indication for ICA, and preprocedural use of beta-blockers and benzodiazepines.

The primary outcome was the NRS anxiety score measured just before the start of the procedure. Secondary outcome measures included NRS anxiety at prespecified time intervals, physiological parameters of stress, patient-reported outcome measures, and provider-reported outcome measures.

NRS anxiety scores were measured at: T0) directly after arrival of the patient at the daycare unit (baseline); T1) 20 minutes prior to transport to the cardiac catheterization room; T2) 20 minutes after T1 and right before transport to the cardiac catheterization room; T3) upon entering the catheterization room and just before the start of the procedure; T4) after arterial puncture, once the coronary catheter was in position (defined as positioning in the aortic root); and T5) after the procedure, when the patient had returned to the daycare unit (Supplemental Figure 2).

Physiological parameters included blood pressure, respiration rate, heart rate, and heart rate variability (HRV), all measured at similar times (T1-T4). The HRV measures were obtained using the Polar H10 chest strap (Polar Electro Oy) and read using EliteHRV (Elite HRV Inc).^20^, ^21^, ^22^ Snapshot measurements of 1 minute were taken with a standard sampling rate of 1,000 Hz. The root mean square of successive differences was extracted as an HRV parameter.

Patient-reported outcome measures in addition to repeated NRS anxiety scores included the State-Trait Anxiety Inventory questionnaire, the Perceived Stress Scale, postprocedural patient satisfaction measured using an NRS scale, and in the intervention group, the Igroup Presence Questionnaire.^23^, ^24^, ^25^, ^26^ Patient-reported VR side effects were registered.

Provider-reported outcome measures comprised cardiologist satisfaction with the procedure using an NRS scale, reported general complexity of the procedure, and complications including radial artery spasm. Patient- and provider-reported outcomes were measured after the procedure (T5). The duration of both VR hypnosis sessions was recorded in minutes.

### Sample size

The power calculation could not be based on previous studies using NRS anxiety scales in patients undergoing ICA, as no such studies have been conducted before. Instead, a 20% difference in NRS anxiety score favoring VR therapy was estimated, based on a previous randomized controlled trial in conscious noncardiac surgery.^27^ With a mean NRS anxiety of 5 in the control group and 4 in the intervention group, and with a SD of 1.5, a sample size of 48 per group was estimated to provide 90% power to detect a significant difference at a 2-sided *P* value of 0.05. Considering potential dropouts based on previous experience with VR in noncardiac surgery studies, 100 patients were included. Patients who did not start the first VR hypnosis session or discontinued participation before the measurements of T1 due to reasons unrelated to the VR intervention (eg, logistical reasons) were excluded from analysis and replaced.

### Statistical analysis

Intention-to-treat and per-protocol analyses were performed for the primary endpoint. For per-protocol analysis, patients were eligible if they completed a minimum of 15 minutes in the first VR hypnosis session and 10 minutes in the second session. The difference in NRS anxiety before arterial puncture (T3) between groups was compared using an independent sample t-test. Secondary analysis of the primary outcome employed analysis of covariance, adjusting for preprocedural benzodiazepine use at the daycare unit and baseline NRS anxiety. A linear mixed model was used to explore possible between-group differences in all repeated outcome measures at all timepoints. The model included “treatment,” “time,” and “treatment-time” as fixed effects, with baseline NRS anxiety values and benzodiazepine use as covariates. Although not originally planned, adjustment for baseline anxiety was performed based on the observation that this covariate was correlated with the primary outcome measure. Beta-blocker use was also added as a covariate in the linear mixed model analysis for the outcome heart rate. Maximum likelihood estimation was used to determine the best-fitting covariance structure through likelihood ratio tests, and restricted maximum likelihood was used for the final model. Residuals were checked for normality, and an alpha of 0.05 was considered significant. Time points T0 to T4 were included in the linear mixed model analysis with repeated measure design. Pairwise comparisons at each time point were conducted if a significant difference was detected. Bonferroni correction was applied to adjust for multiple testing. Linear mixed model analyses were reported as F-ratios with significance levels. The F-ratio represents the ratio of the variation explained by the model and the variation explained by other factors. Therefore, the degrees of freedom of the fixed effects (numerator and denominator) are reported with the F-ratio. Other outcomes were compared using independent sample t-tests or chi-square tests, depending on the data type. Tests were performed 2-tailed, and the results were considered statistically significant when *P* < 0.05.

Prespecified subgroup analyses included analyses according to sex, indication for ICA (elective, NSTE-ACS, and CFT), and VR distraction use between T0 and T1. Benzodiazepine use was included as a covariate in all secondary analyses.

## Results

### Study population

**Figure 1:**
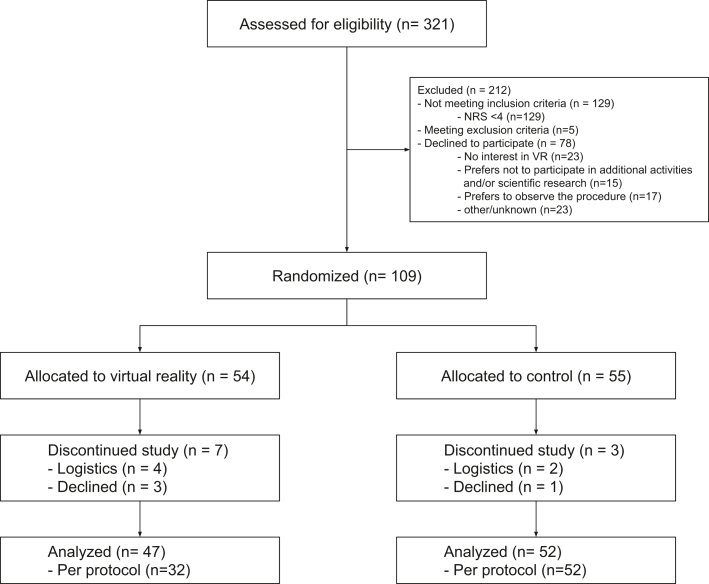
Study Flowchart NRS = numeric rating scale; VR = virtual reality.

Between September 2023 and April 2024, 321 patients were screened. Of these patients, 129 (40%) did not meet inclusion criteria, 78 (24%) declined to participate, and 5 (0.02%) patients met an exclusion criterion, resulting in 109 patients being randomized (Figure 1). Ten patients were replaced, resulting in a total of 99 patients for analysis. Of the 5 patients meeting an exclusion criterion, 3 were excluded due to inability to use VR. Of the 78 patients who declined to participate, 40 patients declined because they would rather not use VR therapy, either because of disinterest or because they preferred to observe the procedure.

The baseline characteristics are shown in Table 1. The mean age (SD) was 67 (±11) years; 55% were females. In the control group, 17 (33%) patients received benzodiazepines at the daycare unit, and 17 (33%) patients at the catheterization room. In the VR group, 10 (21%) patients received benzodiazepines at the daycare unit, and 11 (23%) patients at the catheterization room. No significant differences were observed in baseline characteristics.

**Table 1:** Patient Characteristics

	Control (n = 52)	VR (n = 47)
Age, mean (SD)	67.3 (12.3)	65.7 (9.8)
Female, n (%)	27 (52)	27 (57)
Indication, n (%)		
Elective ICA	39 (75)	33 (70)
Coronary functional test	7 (14)	8 (17)
NSTE-ACS	6 (12)	6 (13)
Previous ICA, n (%)	23 (44)	25 (54)
Previous complications from previous ICA, n (%)	1 (4)	2 (8)
Beta-blocker use, n (%)	33 (64)	30 (64)
Benzodiazepine use, n (%)		
Daycare unit	17 (33)	10 (21)
Catheterization room	17 (33)	11 (23)
STAI trait (range 20-80), mean (SD)	38.3 (8.4)	40.9 (8.9)
STAI state (range 6-24), mean (SD)	13.6 (2.7)	14.1 (2.9)
Perceived stress score (range 0-40), mean (SD)	15.9 (3.9)	17.3 (4.2)

ICA = invasive coronary angiography; NSTE-ACS = non-ST-elevation acute coronary syndrome; STAI = State-Trait Anxiety Inventory; VR = virtual reality.

The mean duration of the first VR hypnosis session was 17.9 (±5.4) minutes. The mean duration of the second VR hypnosis session was 12.6 (±7.2) minutes. Two patients did not complete the first VR hypnosis session, both due to side effects. Three patients did complete the first VR hypnosis session but did not start the second, 2 of whom due to side effects of VR therapy. The reported side effects were nausea, dizziness, and/or headache. Two patients explicitly requested to continue VR hypnosis use after the second session. In consultation with the cardiologist, this was allowed. Their data were included in the analysis as the extended use occurred after the primary outcome was measured, and it was not expected to significantly affect the T5 measurements. The duration of VR hypnosis after the second session was not registered. In the VR group, 24 (51%) patients used VR distraction between T0 and T1, with a mean (SD) duration of 9.8 (4.7) minutes. In all, 4 (9%) patients experienced side effects from VR, of whom 2 reported a headache and 2 reported nausea/dizziness.

### Primary outcome

**Figure 2:**
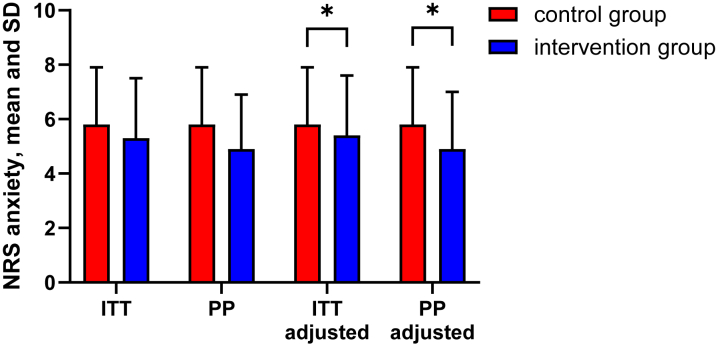
Mean and SD for Numeric Rating Scale Anxiety ITT and PP analysis with and without adjusting for baseline anxiety and benzodiazepine use; ∗*P* < 0.05. ITT = intention to treat; NRS = numeric rating scale; PP = per protocol.

No significant difference was found in (unadjusted) mean NRS anxiety score at T3 (prior to arterial puncture) between control and VR groups in intention-to-treat analysis (mean difference: 0.4; 95% CI: −0.4 to 1.3; *P* = 0.304) and per-protocol analysis (mean difference: 0.9; 95% CI −0.2 to 1.8; *P* = 0.055) (Figure 2). After adjusting for baseline anxiety (T0), significant differences were found in the NRS anxiety score at T3 in favor of the VR group in intention-to-treat analysis (adjusted mean difference: 0.8; 95% CI: 0.1-1.5; *P* = 0.030) and per-protocol analysis (adjusted mean difference: 1.1; 95% CI: 0.4-1.8; *P* = 0.004).

Using linear mixed models, a significant treatment-time interaction was found for NRS anxiety, F (3,266.929) = 4.632, *P* = 0.004 (Table 2). Pairwise comparisons confirmed significant differences at T3 in favor of the VR group (adjusted mean difference: 1.4; 95% CI: 0.7-2.1; *P* < 0.001) using an alpha of 0.0125. No significant differences were observed at other time points.

**Table 2:** NRS Anxiety and Physiologic Anxiety Parameters Over Time

Outcome	T0^a^		T1^a^		T2		T3		T4		*P* Value^b^
	Control (n = 50)	VR (n = 46)	Control (n = 50)	VR (n = 44)	Control (n = 45)	VR (n = 45)	Control (n = 51)	VR (n = 47)	Control (n = 51)	VR (n = 47)	
NRS anxiety	5.7 (1.4)	6 (1.8)	5.4 (1.8)	5.1 (1.7)	5.1 (1.9)	4.2 (2.2)	5.8 (2.1)	5.3 (2.2)	5.4 (2.2)	5.2 (2.4)	0.004
MAP	98 (14)	100 (13)	97 (14)	98 (13)	98 (14)	97 (14)	111 (12)	108 (14)	94 (13)	94 (16)	0.296
Heart rate	71 (11)	70 (14)	68 (11)	69 (12)	68 (11)	69 (13)	73 (13)	71 (14)	74 (15)	71 (15)	0.332
Respiratory rate	15 (3)	15 (5)	14 (3)	15 (5)	14 (3)	13 (5)	15 (3)	14 (5)	14 (4)	13 (3.6)	0.177
RMSSD	33 (29)	35 (30)	37 (33)	39 (40)	43 (48)	33 (42)	32 (23)	35 (29)	33 (38)	32 (33)	0.182

Values are mean (SD). Number of patients is reported for NRS anxiety.

RMSSD (a measure of heart rate variability) is reported in milliseconds.

MAP = mean arterial pressure; NRS = numeric rating scale; RMSSD = root mean square of successive difference; VR = virtual reality.

a T0 and T1 coincided in 9 patients. These measures were duplicated to produce both T0 and T1.

b The *P* value indicated whether there are significant differences between any of the timepoints (T0 to T4), as determined using linear mixed models.

### Secondary outcomes

**Figure 3:**
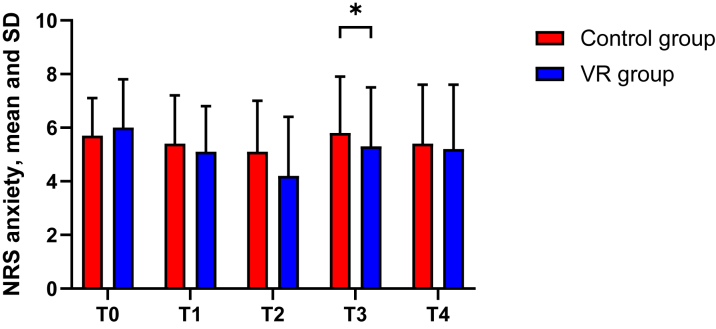
Mean and SD for Numeric Rating Scale Anxiety Over Time Intention-to-treat analysis; ∗*P* < 0.01. Abbreviations as in Figure 1.

No significant difference over time between groups was found for mean arterial pressure (F [3,88.684] = 1.250, *P* = 0.296), respiratory rate (F [3,95.727] = 1.678, *P* = 0.177) (Figure 3), and root mean square of successive differences (F [3,92.130] = 1.656, *P* = 0.182). Similarly, no significant difference was found for heart rate (F [3,128.853] = 1.146, *P* = 0.332) when beta-blocker use was included as a covariate (Table 2).

Patient- and provider-reported outcome measures are shown in Table 3. NRS pain scores for attaining arterial access were not significantly different between the control group (mean: 2.5, SD: 2.7) and VR group (3.0 [2.7], mean difference: −0.5; 95% CI: −1.5 to 0.6; *P* = 0.371). No significant differences were found in the outcomes measured at T5.

**Table 3:** Patient- and Provider-Reported Outcomes at T5

	Control (n = 52)	VR (n = 47)	Mean Difference (95% CI)	*P* Value
NRS anxiety, mean (SD)	3.1 (2.4)	2.9 (2.7)	0.2 (−0.8 to 1.3)	0.641
STAI state, mean (SD)	11.7 (1.9)	12.2 (2.6)	−0.5 (−1.4 to 0.4)	0.251
NRS satisfaction patient, mean (SD)	8.7 (1.1)	8.7 (1.0)	0.03 (−0.4 to 0.4)	0.904
Future VR use, yes (%)	-	35 (75)	n/a	n/a
NRS satisfaction cardiologist, mean (SD)	8.5 (1.3)	8.0 (1.6)	0.5 (−0.1 to 1.1)	0.092
Complexity procedure, n (%)				
Less complex than expected	10 (19)	4 (9)	n/a	0.126
As expected	33 (64)	31 (66)	n/a	0.795
More complex than expected	6 (12)	10 (21)	n/a	0.189
Radial artery spasms (%)	9 (17)	7 (15)	n/a	0.784
Conversion to femoral access (%)	2 (4)	4 (9)	n/a	0.418
Any other complications (%)	2 (4)	4 (9)	n/a	0.423

N/a = not applicable; other abbreviations as in Tables 1 and 2.

Patients in the VR group scored their overall sense of presence, mean (SD), as 3.3 (1.1) on a scale from 0 to 6. Experienced realism was scored 3.4 (1.1), spatial presence 3.5 (1.0), and involvement 3.1 (1.6), all on a scale from 0 to 6. Out of 45 patients, 35 patients (75%) wished to use VR therapy again in the case they had to undergo ICA again in the future.

### Subgroup analyses

No differences were observed in the effect of VR therapy between males and females, as determined by factorial analysis of variance, F (1,94) = 0.000, *P* = 0.987.

No differences in NRS anxiety scores were observed between the use of VR hypnosis alone and VR hypnosis with additional VR distraction between T0 and T1, as determined by one-way ANOVA, F (1,44) = 0.031, *P* = 0.862.

Differences were significant regarding the effect of VR therapy between different indications for ICA, as determined by factorial ANOVA, F (2,92) = 3,720, *P* = 0.028. The effect of VR therapy was only modest in patients who underwent elective ICA or CFT. Patients who underwent ICA for NSTE-ACS experienced a greater effect of VR therapy. Patients undergoing ICA for NSTE-ACS who received usual care experienced an increase in NRS anxiety of 1.1 (95% CI: −0.6 to 3.0) points from baseline to T3, while patients in the VR group experienced a decrease of 1.4 (95% CI: 0.7-2.1) points (Supplemental Table 1).

## Discussion

VR therapy did not reduce preprocedural anxiety. However, VR therapy effectively reduced preprocedural anxiety in patients undergoing ICA, taking into account their baseline anxiety levels (Central Illustration). Patients undergoing ICA for NSTE-ACS seemed to benefit the most from VR therapy. Three-quarters of the patients expressed a desire to use VR therapy again for future procedures. When well-implemented, VR therapy presents an interesting addition to, and possibly an alternative for, benzodiazepines for managing preprocedural anxiety.

**Central Illustration:**
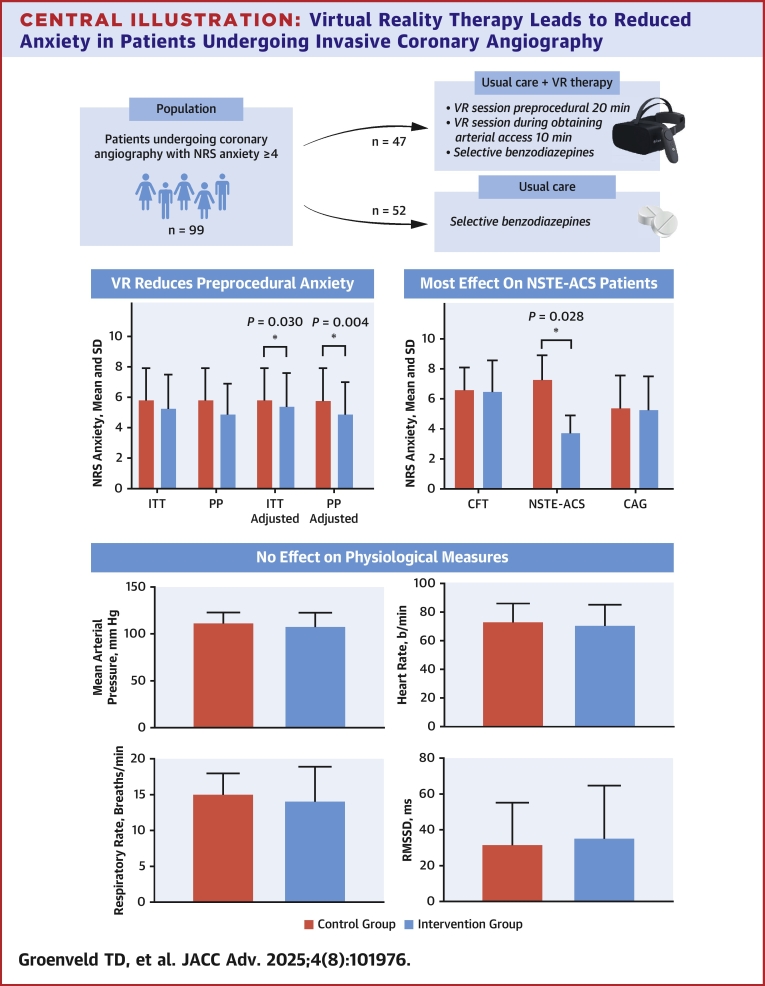
Virtual Reality Therapy Leads to Reduced Anxiety in Patients Undergoing Invasive Coronary Angiography ∗*P* < 0.05. CAG = coronary angiography; CFT = coronary function test; NSTE-ACS = non-ST-segment elevation-acute coronary syndrome; RMSSD = root mean of successive difference; other abbreviations as in Figures 1 and 2.

### Outcomes

In this study, VR therapy reduced preprocedural anxiety by 0.9 to 1.2 points after adjusting for baseline anxiety levels. Although not originally planned, this adjustment was made because this covariate was correlated with the primary outcome measure.^28^ Following CONSORT guidelines, no significance tests were performed for baseline differences in this randomized controlled trial. However, chance differences in baseline variables can still influence the measured effect. It is recommended to assess the relationship between a covariate and the outcome to serve as an indicator of its potential contribution. In this study, baseline anxiety was associated with the primary outcome measure. Baseline NRS anxiety score logically impacts the effect due to known large interindividual variability and its influence on subsequent NRS anxiety scores.

In the evaluation of other nonpharmacological interventions for relieving anxiety around cardiac procedures, a decrease in NRS anxiety of 1 point has been regarded as clinically relevant.^29^ Consequently, the effect of VR therapy can be regarded as clinically relevant. Additionally, the effect found in this study was obtained with mild and transient side effects in only 8% (4/54) of patients, showing that VR therapy is a safe addition to usual care. Patients undergoing ICA for NSTE-ACS experienced a greater effect from VR therapy compared to those undergoing elective ICA. This difference may be attributed to a potentially higher uncertainty of the patient at the preprocedural phases as to what the findings of the angiography procedure may be and potential outcomes. In addition, the preprocedural preparation process is shorter and frequently includes transfers from other hospitals. This leaves less time for thorough patient preparation, including explanation, which has proven to reduce anxiety.^11^^,^^30^ All this in contrast to patients undergoing elective ICA, who have abundant preparation time and are more likely to have already received anxiety interventions. Interestingly, NSTE-ACS patients in the VR group had a lower baseline anxiety score compared to those in the control group, indicating that the observed effect cannot completely be attributed to a higher baseline anxiety level commonly associated with more acute presentations. Further explorations of patients' concerns about the procedure and their expectations of VR therapy may elucidate the reasons behind the differing effect observed between acute and elective ICA procedures.

The effect of VR therapy on the reported NRS anxiety levels was not supported by the physiological measures in this study. Heart rate and HRV are usually the most reactive physiological measures regarding anxiety and have been shown previously to be influenced by VR hypnosis.^31^ However, these measures are also susceptible to medication. Beta blockers, commonly used in the cardiac population as in this study, are known to raise baseline HRV and stabilize both heart rate and HRV at rest.^32^ Concurrently, beta blockers diminish the stress-reactive component of the sympathetic nervous system, resulting in a less pronounced stress-associated decrease in HRV.^33^ This study was not sufficiently powered to perform subgroup analyses on medication interactions, which might be a valuable focus for future research.

### Previous studies

Previous studies have primarily used the State-Trait Anxiety Inventory to measure patient anxiety. In this study, the NRS anxiety score was chosen instead, as it is better suited for a repeated measures design. Additionally, the NRS is quicker to administer repeatedly and likely more sensitive to changes over short intervals. Our results align with those of Keshvari et al, demonstrating the benefits of VR therapy.^34^ However, our study involved a broader patient population, including patients who had undergone previous ICA and those using sedatives, making our findings more generalizable to a wider patient group. The primary endpoint in the Keshvari et al study was assessed immediately postintervention, whereas this study assessed the primary endpoint just before the procedure, which may be more relevant due to the impact of preprocedural anxiety on procedure outcomes.^7^ Gullo et al assessed the effectiveness of VR hypnosis in patients undergoing peripheral vascular intervention and reported a significant difference in preprocedural to postprocedural anxiety reduction in favor of the VR group, aligning with the findings of Keshvari et al.^35^ However, our study found no differences between groups postprocedurally. The intraprocedural use of VR hypnosis in the trials by Keshvari et al and Gullo et al may have contributed to these more pronounced postprocedural effects. In our study, VR therapy was generally discontinued after catheter introduction due to the necessary communication between the cardiologist and patients during coronary angiography, particularly in cases involving coronary function testing. However, in some instances, patients explicitly requested to continue, and this was permitted. Intraprocedural VR therapy might be a valuable addition in cases where less communication between the cardiologist and patient is needed. Unique in this study is the cardiologist perspective explored, which showed equal satisfaction with the procedure in both groups. The cardiologists had to adapt to a slightly altered way of working, minimizing disturbances to the patient during the VR session and allowing them to remain immersed. As with all changes, this initially presented some difficulties. However, by the end of the study, nearly all cardiologists were very positive about the use of VR therapy. Besides effectiveness, considering such provider perspectives is crucial for successful future implementation.

### Strengths and limitations

An important strength of this study is that it comprised an extensive and well-integrated VR protocol. Prior to the study's implementation, health care providers were interviewed on their needs for a successful implementation of a VR intervention. Furthermore, there was a dedicated research member available for all research measurements to prevent any delays in the usual clinical flow due to the required measurements. Another strength is the broad patient population, including different indications for ICA (elective ICA, CFT, and NSTE-ACS). Unfortunately, the subgroups of patients undergoing ICA for CFT and NSTE-ACS were relatively small for performing subgroup analysis. Inclusion of these groups was challenging due to the worldwide unavailability of the acetylcholine necessary for CFT and the short preparation time for patients with NSTE-ACS, which left insufficient time for screening and obtaining informed consent. The subgroup analyses should therefore be interpreted with caution due to the small group sizes. Another limitation of this study is that patients' expectations regarding the procedure and VR therapy were not assessed. Outcome expectations and patient-related self-efficacy expectations are known to influence the effects of medical treatment.^36^ Exploring these expectations could provide deeper insights into why the effectiveness of VR therapy varies significantly between patient groups and among individual patients.

### Implications for clinical practice

This study demonstrates that VR therapy can be a valuable addition to the usual care with benzodiazepines for managing preprocedural anxiety in patients undergoing ICA. VR therapy might also serve as a possible nonpharmacological alternative for patients who have contraindications to benzodiazepines, those who refuse to take benzodiazepines, or in cases where the ICA protocol prohibits benzodiazepine use. Outside of research settings, VR therapy can be administered easily and on demand, including when anxiety arises during the preparation phase or throughout the procedure. The inclusion criteria in this study, particularly the NRS anxiety score of 4 or more, were designed to ensure measurable effectiveness and relevance to the studied population. However, in clinical practice, these criteria could be applied more flexibly. NRS scores vary significantly between individuals and may not fully capture subjective anxiety levels. For example, some patients with lower NRS scores during screening still expressed interest in VR, indicating its potential for broader use. Patients excluded due to the inability to use VR represent a small group unlikely to benefit in practice. A more notable barrier was patients declining participation due to study-related procedures, who might accept VR therapy when offered without these burdens. Additionally, hesitancy toward new technology like VR is expected to decrease as it becomes more familiar.

The results of this study may pave the way for using VR therapy in other diagnostic or therapeutic procedures including electrophysiology and percutaneous valve procedures, where anxiety can impact the patient outcome and effectiveness of the procedure. Furthermore, many procedures are performed under sedation due to severe patient anxiety or the need for patients to remain still for extended periods. Preprocedural VR therapy may alleviate anxiety beforehand, allowing patients to approach such procedures more relaxed. More research is needed to evaluate the effect of extended VR therapy during procedures, ultimately investigating its viability as an alternative to sedation for anxious patients. Of particular interest would be a trial including (a larger group of) patients undergoing emergency angiography, as the patients in the NSTE-ACS group seem to benefit most from VR therapy. As health care is evolving toward patient-centered care models, incorporating innovative approaches like VR therapy can minimize procedural distress, significantly enhance patient comfort, and improve overall health care experiences.

## Conclusions

VR therapy did not reduce preprocedural anxiety. However, VR therapy effectively reduced preprocedural anxiety in patients undergoing ICA when adjusting for baseline anxiety, and the effect seems most pronounced in patients undergoing ICA for NSTE-ACS. VR therapy can serve as a valuable nonpharmacological addition or possible alternative to pharmacological treatment of procedure-related anxiety.

Perspectives **COMPETENCY IN PATIENT CARE AND PROCEDURAL SKILLS:** This study demonstrates that VR therapy can complement usual care in managing preprocedural anxiety in patients undergoing ICA and may serve as a nonpharmacological alternative when benzodiazepines are contraindicated or restricted. Easily administered on demand, VR therapy could potentially benefit other anxiety-prone procedures, such as electrophysiology and percutaneous valve interventions. Implementation barriers will likely include staff training and integration into existing workflows—both crucial for the successful adoption and effectiveness of VR therapy in clinical practice.

**TRANSLATIONAL OUTLOOK:** Further research should explore its intraprocedural use and potential as a sedation alternative, particularly in emergency angiography, given the benefits observed in NSTE-ACS patients.

## Funding support and author disclosures

The authors have reported that they have no relationships relevant to the contents of this paper to disclose.
