# Supplementary material for: Virtual Reality to Reduce Preprocedural Anxiety During Invasive Coronary Angiography: The VR InCard Trial
Source: JACC Adv. 2025 Jul 18;4(8):101976. doi: 10.1016/j.jacadv.2025.101976 (PMC12301777; doi:10.1016/j.jacadv.2025.101976)
Supplement: Supplementary data [file mmc1.pdf]

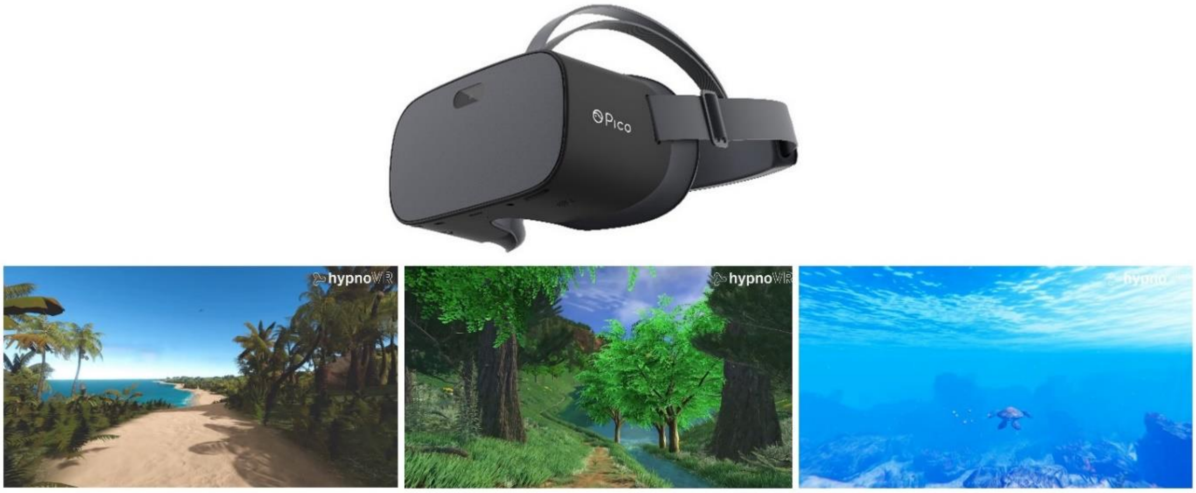

Supplemental Figure 1. Pico G2 4K headset and examples of available environments in HypnoVR.

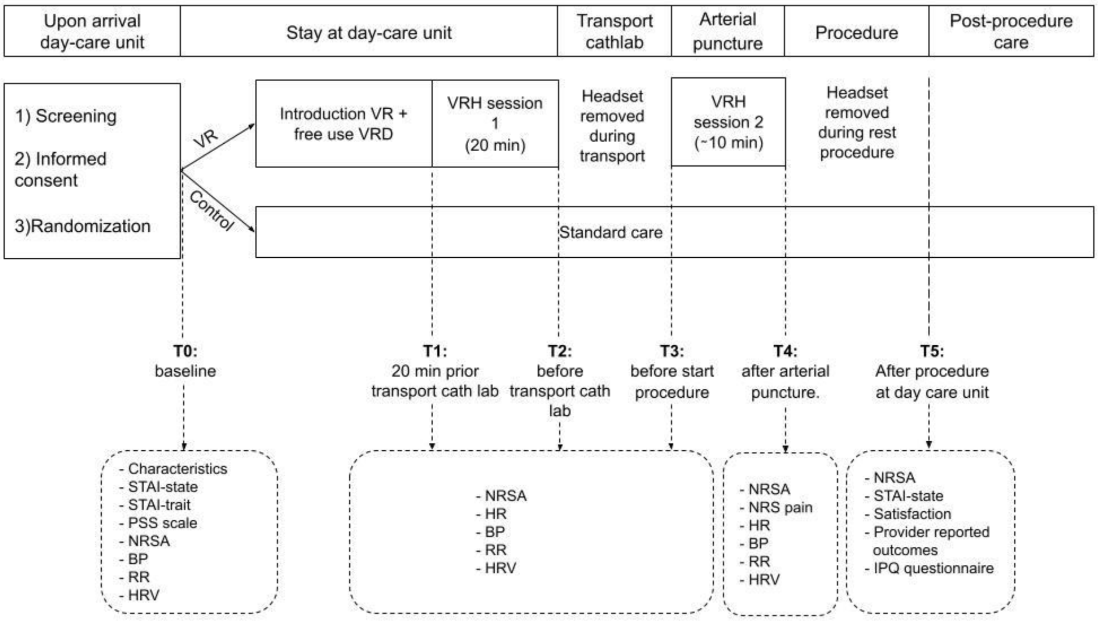

Supplemental Figure 2. Study procedures and measurements. STAI – State Trait Anxiety Inventory; PSS –Perceived Stress Scale; NRSA – Numeric Rating Scale anxiety; BP – blood pressure; RR – respiratory rate; HRV – heart rate variability; IPQ – Igroup Presence Questionnaire. VRH – Virtual reality hypnosis; VRD – virtual reality distraction.

Supplemental Table 1. NRS anxiety by indication and treatment

Mean (SD)	T0		T3	
	Control	VR	Control	VR
CFT	5.6 (1.3)	6.4 (1.8)	6.6 (1.5)	6.5 (2.1)
NSTE-ACS	6.2 (1.7)	5.0 (1.2)	7.3 (1.6)	3.8 (1.1)
Elective ICA	5.6 (1.4)	6.1 (1.9)	5.4 (2.2)	5.3 (2.2)

NRS – Numeric Rating Scale; CFT – coronary function testing; NSTE-ACS – non ST-elevation acute coronary syndrome; ICA – invasive coronary angiography; VR- virtual reality.
